# The effect of transcutaneous auricular vagus nerve stimulation on cycling ergometry and recovery in healthy young individuals

**DOI:** 10.1002/brb3.3332

**Published:** 2023-11-16

**Authors:** Sefa Haktan Hatik, Mesut Asrlan, Ömer Demirbilek, Ali Veysel Özden

**Affiliations:** ^1^ Department of Health Care Services, Türkeli Vocational School Sinop University Sinop Turkey; ^2^ Physiotherapy and Rehabilitation Department, Health Sciences Faculty Bitlis Eren University Bitlis Turkey; ^3^ Department of Emergency and Disaster Management, Türkeli Vocational School Sinop University Sinop Turkey; ^4^ Physiotherapy and Rehabilitation Department, Health Sciences Faculty Bahçeşehir University Istanbul Turkey

**Keywords:** auricular, cycling ergometry, recovery, transcutaneous, vagus nerve stimulation

## Abstract

**Background:**

It is aimed to examine the potential benefits and effects of the use of transcutaneous auricular vagus nerve stimulation (VNS) for sporting purposes on recovery, fatigue, and sportive performance level.

**Methods:**

In this study, 90 people between the ages of 18–23 were participated. They were randomly divided into three groups as bilateral sham, unilateral left, and bilateral VNS. A 4‐day protocol was applied to the participants. Cycling exercise was performed with maximum performance for 30 min under the same watt load. Pulse, systolic and diastolic blood pressure, distance, pain, fatigue, lactic acid level, and autonomic nervous system were evaluated.

**Results:**

Within the groups, there was a statistically significant difference between the data (*p* < .05) except for the distance covered parameter. When we compare the groups, in addition to the distance traveled in all groups, there is no statistically significant difference in the 1st day 1st measurement and 2nd measurement data of all parameters (*p* > .05 When we compared the data according to days, there was a statistically significant difference between bilateral stimulation (BS) and unilateral stimulation, only pain and fatigue levels (*p* < .05).

**Conclusion:**

In our study, we saw that BS application gave positive results in reducing pain and fatigue due to cycling exercise compared to other applications. Similar results were obtained when we evaluated the data on a daily basis. We believe that VNS will be beneficial in reducing pain and fatigue, especially during and after the competition halftime.

## Graphical abstract

It can be said that our research is a pioneering study by evaluating the sportive effects of vagus nerve stimulation. It shows that especially bilateral application can improve physiological parameters (such as blood lactic acid level, blood pressure and pain) related to sports activity together with tonomic nervous system changes. Bilateral application can be a promising technique to improve sports activities (performance and recovery) by its noninvasiveness and without doping and apparent side effects.

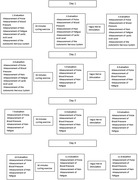

## 1 INTRODUCTION

In our bodies, the structure that controls the functions of the internal organs involuntarily is the autonomic nervous system. It has a key role in maintaining homeostasis. Generally, the sympathetic system regulates catabolic events, whereas the parasympathetic system regulates anabolic events (McCorry, 2007). The vagus nerve is the most innervating cranial nerve in the body. This nerve carries sensory, motor, and parasympathetic information. The task of the nerve is to activate the parasympathetic system and support the “rest and digest” process to provide homeostasis (Karemaker, 2017; Koopman et al., 2011).

Stimulation of the vagus nerve can be done manually or electrically. For manual stimulation of the vagus nerve, massage and compression are applied on the carotid artery in the neck region. Later, with the development of technology, electrical stimulation methods began to be used. In the method that uses electrical stimulation, invasive and noninvasive applications are made. Noninvasive applications are simple and reduce the risk of infections and other surgery complications (Howland, 2014; Lanska, 2002). Noninvasive vagus nerve stimulation (VNS) can reduce sympathetic activity and can improve cardiac baroreflex sensitivity and autonomic modulation (Antonino et al., 2017; Clancy et al., 2014). Right or left sided auricular vagus nerve afferent fibers terminate at the same nuclei in the brain stem and then the stimulus effect is dispersed. As the stimulation is processed in the brainstem, right and left ear stimulation is cardiac‐safe (Chen et al., 2015; Murray et al., 2016).

Today, with the professionalization of sports as a result of globalization, sports activities have lost their characteristics of being games. For this reason, the capitalist economic order has dominated sports. Football, one of the most popular sports branches, has become a multimillion dollar industry with revenues such as broadcasting, advertising, sponsorship, and matchdays (Galariotis et al., 2018; Uhrich, 2021). With the industrialization of sports, more competition means more income. In addition, during these competitions, athletes are required to perform at a high level. For these reasons, sports clubs and athletes are looking for different methods in order to reduce the level of fatigue in a short time and accelerate recovery.

With the start of exercise or sports activity, sympathetic activity increases in the body and reaches the plateau value in maximal activity after a certain period. With the end of sports activity, the suppressed parasympathetic activity starts to increase, and the sympathetic system returns to the resting state in time (Coote, 2010). After exercise, parasympathetic system activation continues for up to 48 h. If the exercise is intense and resistant, parasympathetic system activity can extend up to 72 h. Moreover, due to anaerobic respiration rate increases during exercise, there may be decreases in parasympathetic reactivation (Buchheit et al., 2007).

Hence, in the light of the literature, in our study, we aimed to examine the potential benefits and effects of the use of transcutaneous auricular VNS for sports purposes, which will be applied due to the high level of performance expectation from the athletes and the increasing number of competitions, on the recovery, fatigue, and sportive performance level of the athletes. We hypothesized that auricular VNS can reduce sympathetic activity, increase parasympathetic activity, and speed up exercise recovery.

## 2 MATERIALS AND METHODS

### 2.1 Design of the study

**FIGURE 1:**
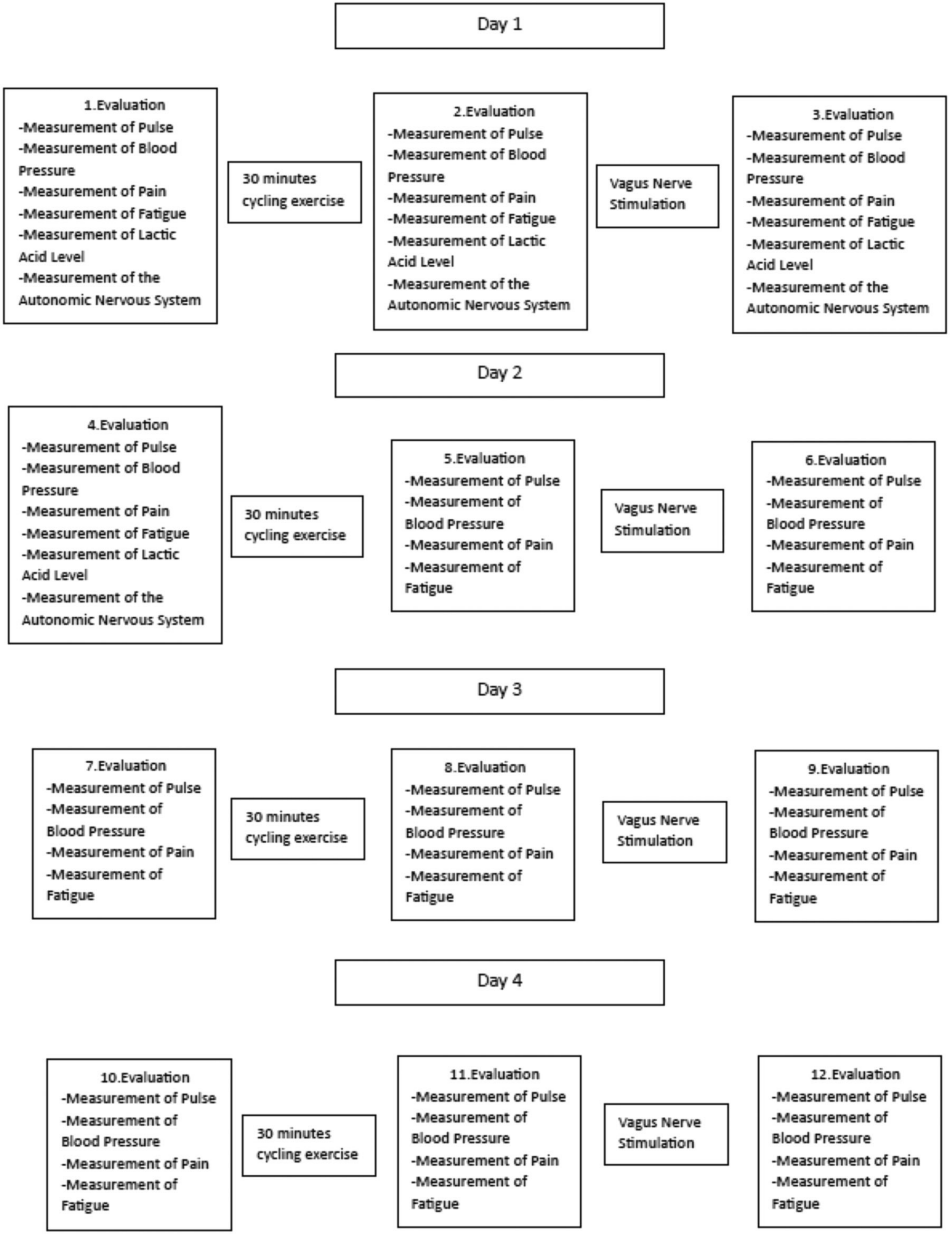
Protocol of the study.

It is a single‐blind randomized clinical trial, including pre‐ and posttest evaluation methods, in which the evaluator was blinded. The details of the study protocol will be explained in detail. Additionally, thisprocess is visualized in detail in Figure 1.

### 2.2 Subjects

In this study, 106 volunteer healthy individuals between the ages of 18–35 participated in the laboratory of Sinop University, Türkeli Vocational School. Inclusion criteria for the study are (1) being healthy between the ages of 18–35, (2) volunteering to participate in the study, (3) signing a voluntary consent form, and (4) being in the inactive category according to the International Physical Activity Questionnaire. Exclusion criteria from the study are (1) having regular sports habits, (2) being pregnant or suspected of pregnancy, (3) disability of the lower or upper extremities, (4) acute wound or infection in the ear, (5) those who are in the very minimally active or active category according to the International Physical Activity questionnaire, (6) presence of any chronic disease and drug use for this condition, (7) current history of respiratory system disease, and receiving treatment, (8) existing cardiac system disease history and receiving treatment, and (9) existing hearing system disease history and receiving treatment.

**FIGURE 2:**
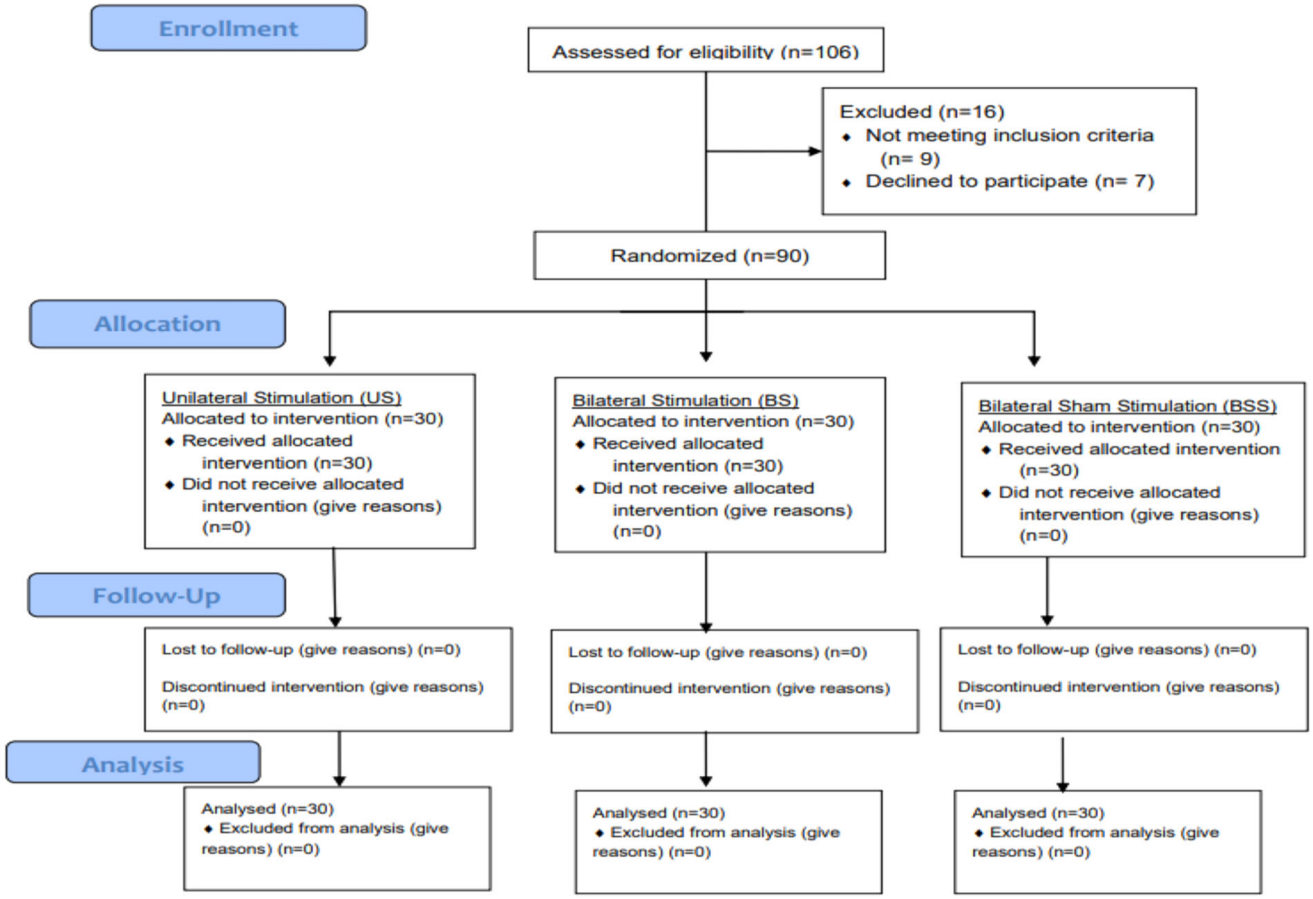
Consort diagram.

After informing the participants in detail about the procedure to be applied, an international physical activity questionnaire was first applied to the volunteers who signed the consent form (Van Poppel et al., 2010). Out of 106 people evaluated for eligibility, 4 people with regular sports habits, 5 people in the active category according to the results of the international physical activity survey, 7 people who refused to participate in the study, and 90 participants were continued (Figure 2). The gender, age, height, weight, and body mass indexes of the participants who met the inclusion criteria were recorded and randomly divided into three groups. No one left the study for any reason after randomization.

Gender, age, height, weight, and body mass index of the participants who met the inclusion criteria were recorded and randomly divided into three groups. The groups are unilateral stimulation (US) group (*n* = 30, 15 women, 15 men), bilateral stimulation (BS) group (*n* = 30, 15 women, 15 men), and bilateral sham group (*n* = 30, 15 women, 15 men).

The study was carried out at Sinop University for 4 days, in a quiet laboratory environment between 10.00 and 12.00, with the room temperature fixed at 25 ± 2°C 1 h before. Participants were limited to alcohol and caffeine intake at least 24 h beforehand. The preovulation period was questioned and applied to the female participants.

A 4‐day protocol was applied in the study. All subjects had their pain, fatigue, pulse, and blood pressure measured before the application on every 4 protocol days. Autonomic nervous system and lactic acid levels were measured only in three evaluation stages on Day 1 and in the first evaluation stage on Day 2. The bicycle ergometer test was performed after the 1st evaluation phase of each day.

After the first evaluation in the study protocol was completed, participants in all groups performed cycling exercise against 25 W resistance for 30 min. After the exercise, the distance covered was noted, and a second daily evaluation was made. After the evaluation, the participants received noninvasive ear VNS for 20 min according to their randomization groups. After the application, the measurement was repeated, and the daily protocol was terminated (Figure 1 ).

### 2.3 Vagus nerve stimulation

**FIGURE 3:**
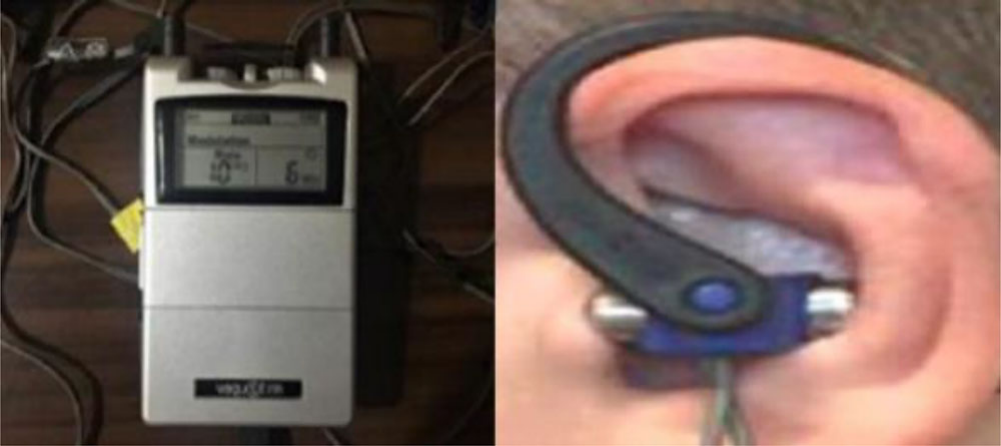
Vagustim device and transcutaneous vagus nerve stimulation.

Application was made to the Bilateral and Unilateral groups in the study. In the sham group, the application was performed with a specially produced headset that does not conduct current. Applications in the unilateral group were performed on the left ear of the subjects. In the bilateral group, the application was performed in both ears. The application was performed for 20 min in all groups. In Bilateral and Unilateral groups, VNS was performed from the Vagustim device (Vagustim) with a frequency of 10 Hz, biphasic, 300 μs pulse width and modulation mode. In the current intensity adjustment, it was adjusted according to the subject's detection threshold and kept constant at this setting. The application was placed in contact with the cymba concha and tragus of the ear through a headset placed in the outer ear. To increase conductivity, a gel was applied to the headphone before application (Figure 3).

### 2.4 Sample size

In order to determine the number of samples, power analysis was performed using the G*Power (v3.1.9.7) program. The power of the study is expressed as 1 − *β* (*β* = probability of type II error). In Liu et al.’s (2017) study, evaluating the heart rate variability (HRV) predicting the response to VNS in patients with drug‐resistant epilepsy, the measurement data of the control group (1064 ± 562) and the data of the epileptic patient group, based on the mean and standard deviation values of LF power that are (608 ± 406), scores were reached . The effect size (*d*) was found to be 0.348 in the calculation made to obtain 80% power at the *α* = .05 level of the study. Accordingly, it was determined that there should be 84 people in total for this study, which has 3 groups.

### 2.5 Randomization

The subjects participating in the study were assigned numbers from 1 to 90. Simple random sampling method was used. A number sequence consisting of 30 numbers was produced for each group from the Microsoft Office Excel program. Groups were formed with the generated number sequence.

### 2.6 Outcome measures

#### 2.6.1 Primary outcome measures

The main settings in the study are the analysis of the autonomic nervous system by utilizing a wearable chest belt and HRV (Perrotta et al., 2017).

#### 2.6.2 Analysis of the autonomic nervous system

**FIGURE 4:**
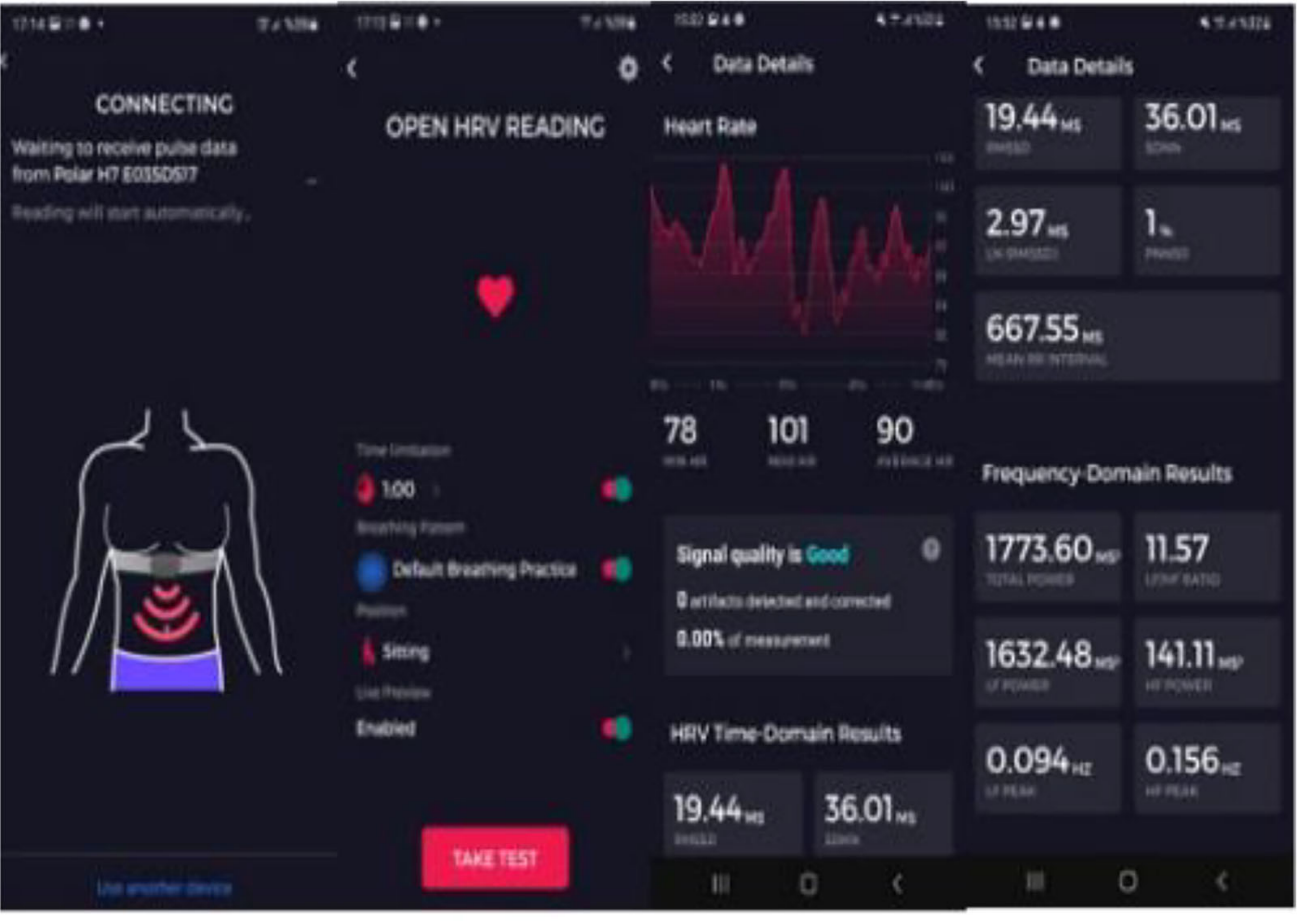
Elite heart rate variability (HRV) software.

In the study, the autonomic nervous system was evaluated with the Polar H10 device. Data was recorded by connecting the device to a smart phone via Bluetooth. Elite HRV software developed by the manufacturer of the device was used to analyze the data (Figure 4). The participant was in a sitting position, and a measurement lasting about 1 min was made. In order for the device to make accurate measurements, the electrode surface was disinfected and wetted before each measurement to increase its conductivity. The subjects were prevented from speaking and moving during the measurement.

The Polar H10 device is an HRV sensor that comes with a wearable chest strap and is the gold standard in high sensitivity and accuracy. It can be connected to multiple training devices via Bluetooth and ANT+. The device comes with a soft, adjustable sensor that contacts the chest to capture HRV in real time. Current evidence for smartphone applications that can sync with a personal monitor to collect R–R intervals via the Elite HRV app has demonstrated user‐friendly software for instant analysis of HRV. Additionally, HRV smartphone applications often offer the option to transfer raw R–R data files directly to the practitioner's computer via email. Elite HRV, a smartphone application, was judged to be a valid platform to examine RMSSD compared to Kubios HRV 2.2, a widely used HRV analysis software (Perrotta et al., 2017).

#### 2.6.3 Secondary outcome measures

Secondary evaluation in the study was used for fatigue level, fatigue rating scale (Micklewright et al., 2017), lactic acid level (Tanner et al., 2010), and pain numerical pain scale (Farrar et al., 2001). Additionally, pulse and blood pressure were measured with a blood pressure device attached to the right wrist.

#### 2.6.4 Lactic acid level measurement

Lactate Scout Plus device was used to evaluate lactic acid level. Lactate Plus (L+, Nova Biomedical) uses an electrochemical lactate oxidase biosensor to measure lactate in whole blood. A blood sample of 0.7 μL is required; Sample analysis time is 13 s. Test strips used with the device do not require calibration codes or special calibration strips. The device is equipped with a two‐level quality control solution that is used before testing to ensure correct operation of the analyzer (Tanner et al., 2010).

Normally, a sample is taken from the earlobe to make a painless measurement. However, as VNS was performed through the ear, samples were taken from the middle finger of the participants’ right hand in order to prevent the effect of measurement from there on autonomic activity. In order to minimize the error rate of the data, the finger to be measured was cleaned with a clean and sterile cotton beforehand.

#### 2.6.5 Numerical fatigue rating scale

It is a one‐dimensional measurement method used to evaluate fatigue. Numbers between 0 and 10 are placed on the visual analogue scale. There are opposite statements about fatigue at the extreme ends of the scale. The measurement is completed by asking the patient to mark the number representing the fatigue level (Micklewright et al., 2017).

#### 2.6.6 Numerical pain rating scale

It is a one‐dimensional method that subjectively evaluates the patient's acute pain level. It uses numbers placed on the visual analog scale: “0” means no pain and “10” means unbearable pain. Evaluation is made by asking the patient to mark his/her current pain level on the scale (Farrar et al., 2001).

#### 2.6.7 Pulse and blood pressure measurement

The participants’ pulse and blood pressure were measured from the right wrist in a sitting position with a Braun BPW4500 blood pressure monitor. The device uses oscillometric method for measurement. The reading range of the device for blood pressure value is 40–255 mmHg, whereas for pulse rate, it is 40–199 bpm. Although the margin of error is ±3 mmHg for blood pressure value, it is ±4% for pulse rate. The device is calibrated during production and does not need to be recalibrated when used in accordance with the instructions. The measurements were automatically recorded in the device's memory and then recorded on the patient follow‐up forms. Although the device is measuring, the subject is in a sitting position and the subject does not move or speak for 60 s.

#### 2.6.8 Bicycle ergometer test

Participants in all groups were asked to exercise for 30 min using a V‐fit X bike magnetic exercise bike with a resistance value of 25 W. After exercise, the distance traveled for 30 min was recorded in kilometers. The aim here was to evaluate performance by comparing the distances traveled against the same resistance force and duration.

#### 2.6.9 Permission

The study was the appropriate plan of the Declaration of Helsinki. Before starting the study, ethics committee approval dated 29.09.2021 and numbered 2021/6 was obtained from Gümüşhane University Scientific Research and Publication Ethics Committee. The trial was registered with the ClinicalTrials.gov registry (Study Identifier: NCT05778058).

#### 2.6.10 Data analysis

While evaluating the findings obtained in the study, IBM SPSS Statistics 29.0 program was used for statistical analysis. The suitability of the parameters to normal distribution was evaluated by examining Skewness and Kurtosis values, histogram and *Q*–*Q* pilot graphs. It was determined that the data showed normal distribution. ANOVA test was used for comparisons between groups as there were three groups. Post hoc analysis was performed to determine the source of the difference. Tukey HSD test was used if the variances of the groups were homogeneous, and Tamhane's T2 test was used if they were not homogeneous. Repeated measure ANOVA test was used for repeated measurements within the group. To determine which measurement caused the difference, the Bonferroni test was used as a post hoc test. The significance level for all tests was accepted as *p* < .05.

## 3 RESULTS

There was no significant difference between the demographic data of the groups before the application (Table 1).

**TABLE 1:** Demographic data of groups.

Parameters	Mean ± SD			*p*
	US	BS	BSS	
**Age**	20.1 ± 1.06	20.4 ± 0.89	20.57 ± 1.55	.317
**Heıght**	1.69 ± 0.1	1.69 ± 0. .08	1.7 ± 0.08	.929
**Weıght**	66.1 ± 13.85	63.6 ± 8.98	63.4 ± 12.91	.630
**BMI**	22.96 ± 4.06	22.16 ± 2.52	21.78 ± 3.26	.377
**F/M**	15/15	15/15	15/15	^+^1.000

*Note*: One‐way ANOVA test, +continuity (yates) correction.

Abbreviations: BS, bilateral stimulation; BSS, bilateral sham stimulation; SD, standard deviation, US, unilateral stimulation.

In the evaluation made after VSS application on all days and at the 24th hour after the application, the fatigue values of the US and BS groups decreased significantly compared to the bilateral sham stimulation (BSS) group. In addition, the fatigue values of the BS group decreased significantly compared to the US group in the evaluation made after the 2nd and 4th day VSS application and at the 24th hour after the 2nd day application. Considering the changes within the group after the vagus application, fatigue values decreased significantly in all groups on all days (Table 2).

**TABLE 2:** Comparison of intragroup, intergroup, and differences in fatigue and pain parameters.

Parameters			Mean ± SD (Median)			^1^ *p*
			US	BS	BSS	
**Fatigue** **(0–10)**	**1st day**	**1st measurement**	0 ± 0 (0)^A^	0 ± 0 (0)^A^	0 ± 0 (0)^A^	1.000
		**2nd measurement**	7.47 ± 1.14 (8)^A^	7.57 ± 0.97 (8)^A^	7.37 ± 1.16 (8)^A^	.778
		**3rd measurement**	4.67 ± 1.03 (5)^A^	3.97 ± 0.81 (4)^A^	6.53 ± 1.04 (6)^B^	<.001
		**^2^ *p***	.001*	.001*	.001*	
		**2nd–3rd**	**2**.**8 ± 0**.**61 (3)**	**3**.**6 ± 0**.**56 (4)**	**0**.**84 ± 0**.**75 (1)**	**<**.**001**
	**2nd day**	**1st measurement (24th hour)**	2.97 ± 0.72 (3)^A^	2.63 ± 0.56 (3)^A^	6.20 ± 1.06 (6)^B^	<.001
		**2nd measurement**	7.87 ± 0.86 (8)^A^	6.77 ± 0.86 (7)^B^	8.03 ± 0.72 (8)^A^	<.001
		**3rd measurement**	4.70 ± 1.09 (4.5)^A^	3.20 ± 0.76 (3)^B^	6.5 ± 0.63 (6.5)^C^	<.001
		**^2^ *p***	.001*	.001*	.001*	
		**2nd–3rd**	**3**.**17 ± 0**.**70 (3)**	**3**.**57 ± 0**.**68 (4)**	**1**.**53 ± 0**.**97 (1)**	**<**.**001**
	**3rd day**	**1st measurement**	2.73 ± 0.69 (3)^A^	2.00 ± 0.53 (2)^B^	3.93 ± 0.94 (4)^C^	<.001
		**2nd measurement**	7.73 ± 0.74 (8)^A^	6.57 ± 0.94 (7)^B^	7.83 ± 0.65 (8)^A^	<.001
		**3rd measurement**	5.20 ± 0.61 (5)^A^	2.83 ± 0.65 (3)^B^	5.47 ± 0.94 (5.5)^A^	<.001
		**^2^ *p***	.001*	.001*	.001*	
		**2nd–3rd**	**2**.**53 ± 0**.**73 (3)**	**3**.**73 ± 0**.**58 (4)**	**2**.**37 ± 0**.**81 (2)**	**<**.**001**
	**4th day**	**1st measurement**	2.67 ± 0.66 (3)^A^	1.77 ± 0.63 (2)^B^	3.03 ± 0.72 (3)^A^	<.001
		**2nd measurement**	7.80 ± 0.71 (8)^A^	6.17 ± 0.91 (6)^B^	8.00 ± 0.98 (8)^A^	<.001
		**3rd measurement**	4.70 ± 0.65 (5)^A^	2.80 ± 0.71 (3)^B^	5.87 ± 1.11 (6)^C^	<.001
		**^2^ *p***	.001*	.001*	.001*	
		**2nd–3rd**	**3.10 ± 0.55 (3)**	**3.37 ± 0.56 (3)**	**2.13 ± 0.97 (2)**	**<.001**
**Pain** **(0–10)**	**1st day**	**1st measurement**	0 ± 0 (0)^A^	0 ± 0 (0)^A^	0 ± 0 (0)^A^	1.000
		**2nd measurement**	7.53 ± 0.51 (8)^A^	7.30 ± 0.47 (7)^A^	7.57 ± 0.73 (8)^A^	.157
		**3rd measurement**	5.43 ± 0.5 (5)^A^	4.27 ± 0.58 (4)^B^	5.93 ± 0.78 (6)^A^	<.001
		**^2^ *p***	.001*	.001*	.001*	
		**2nd–3rd**	**2**.**10 ± 0**.**48 (2)**	**3**.**03 ± 0**.**61 (3)**	**1**.**63 ± 0**.**89 (2)**	**<**.**001**
	**2nd day**	**1st measurement (24th hour)**	3.57 ± 0.63 (3.5)^A^	2.90 ± 0.8 (3)^B^	4.37 ± 0.81 (4)^C^	<.001
		**2nd measurement**	6.97 ± 0.67 (7)^A^	6.63 ± 0.85 (7)^A^	6.97 ± 0.67 (7)^A^	.133
		**3rd measurement**	4.90 ± 0.71 (5)^A^	3.37 ± 0.49 (3)^B^	5.57 ± 0.63 (5.5)^C^	<.001
		**^2^ *p***	.001*	.001*	.001*	
		**2nd–3rd**	**2**.**07 ± 0**.**58 (2)**	**3**.**27 ± 0**.**91 (3)**	**1**.**40 ± 0**.**72 (1)**	<.001
	**3rd day**	**1st measurement**	3.53 ± 0.51 (4)^A^	2.57 ± 0.5 (3)^B^	4.20 ± 0.66 (4)^C^	<.001
		**2nd measurement**	7.30 ± 0.47 (7)^A^	5.33 ± 0.61 (5)^B^	7.17 ± 0.70 (7)^A^	<.001
		**3rd measurement**	5.40 ± 0.50 (5)^A^	3.03 ± 0.72 (3)^B^	5.87 ± 0.63 (6)^A^	<.001
		**^2^ *p***	.001*	.001*	.001*	
		**2nd–3rd**	**1**.**90 ± 0**.**61 (2)**	**2**.**30 ± 0**.**88 (2)**	**1**.**30 ± 0**.**65 (1)**	<.001
	**4th day**	**1st measurement**	3.53 ± 0.51 (4)^A^	2.40 ± 0.50 (2)^B^	4.13 ± 0.73 (4)^A^	<.001
		**2nd measurement**	6.93 ± 0.69 (7)^A^	4.47 ± 0.57 (4)^B^	6.83 ± 0.75 (7)^A^	<.001
		**3rd measurement**	5.20 ± 0.71 (5)^A^	2.40 ± 0.62 (2)^B^	5.37 ± 0.49 (5)^A^	<.001
		**^2^ *p***	.001*	.001*	.001*	
		**2nd–3rd**	**1**.**73 ± 0**.**52 (2)**	**2**.**07 ± 0**.**94 (2)**	**1**.**47 ± 0**.**57 (1**.**5)**	.006

*Note*: ANOVA test, repeated measure ANOVA test. Different capital letters in the lines indicate the difference between groups.

Abbreviations: BS, bilateral stimulation; BSS, bilateral sham stimulation; SD, standard deviation, US, unilateral stimulation.

*p < .05.

The pain scores of the BS group decreased significantly compared to the US and BSS groups in the evaluation made after the 1st, 3rd, and 4th day VSS application and at the 24th hour after the 3rd day application. In the evaluation made after the 2nd day VSS application and at the 24th hour after the 1st day/2nd day application, the pain scores of the US and BS groups decreased significantly compared to the BSS group, whereas the pain scores of the BS group decreased significantly compared to the US group. Considering the changes within the group after the VSS application, pain scores decreased significantly in all groups on all days (Table 2).

The difference in the numerical fatigue value among the 1st day, the 2nd measurement, and the 3rd measurement shows a statistically significant difference between the groups. The largest decrease among the 1st day, 2nd, and 3rd measurement was seen in the BS group (*p* < .001). The numerical fatigue value difference between the 2nd and the 3rd measurements on the 2nd day shows a statistically significant difference between the groups. The largest decrease among the 2nd day, 2nd, and 3rd measurement was seen in the BS group (*p* < .001). When the numerical fatigue values were examined, the highest decrease between the 2nd and 3rd measurements was seen in the BS group in the measurements made in all 4 days. Similarly, when numerical pain values were examined, the highest decrease between the 2nd and 3rd measurements in the measurements made in 4 days was seen in the BS group. The decrease between the 2nd and 3rd measurement shows a statistically significant difference between the groups (*p* < .001) (Table 2).

After the VSS application, the lactic acid values of the BS group decreased significantly compared to the US and BSS groups. However, there was no significant difference between the other groups and in the evaluation made at the 24th hour after the application. Considering the intragroup changes in the evaluation made after the VSS application and at the 24th hour after the application, lactic acid values decreased significantly in all groups. No significant difference was found in the distance parameter in both between‐ and in‐group analyses (Table 3).

**TABLE 3:** Comparison of intragroup, intergroup, and differences in lactic acid level and distance parameters.

Parameters		Mean ± SD (median)			^1^ *p*
		US	BS	BSS	
**Lactic acid level**	**1st measurement**	3.66 ± 2.05 (3.7)^A^	3.59 ± 2.26 (2.7)^A^	3.73 ± 1.72 (3.5)^A^	.968
	**2nd measurement**	12.77 ± 4.67 (12.1)^A^	13.53 ± 4.63 (12.1)^A^	12.89 ± 2.89 (13)^A^	.746
	**3rd measurement**	7.94 ± 4.10 (7)^A^	5.56 ± 2.54 (5.2)^B^	9.62 ± 2.61 (9.9)^A^	<.001
	**4th measurement (24th hour)**	2.91 ± 1.74 (2.5)^A^	3.07 ± 2.16 (2.3)^A^	3.41 ± 1.98 (3.3)^A^	.609
	**^2^ *p***	.001*	.001*	.001*	
	**2nd–3rd**	**4**.**83 ± 0**.**75 (4**.**85)**	**4**.**85 ± 2**.**88 (7**.**2)**	**3**.**27 ± 0**.**59 (3**.**2)**	**<**.**001**
	**3rd–4th**	**5**.**03 ± 4**.**50 (−3**.**05)**	**2**.**49 ± 3**.**01 (−2**.**25)**	**6**.**21 ± 3**.**08 (−5**.**95)**	**<**.**001**
**Distance**	**1st measurement**	13.12 ± 3.18 (13.1)^A^	13.30 ± 3.98 (12.4)^A^	13.15 ± 2.79 (12.4)^A^	.975
	**2nd measurement**	13.23 ± 3.82 (12.6)^A^	13.73 ± 4.81 (11.7)^A^	13.11 ± 3.60 (12)^A^	.827
	**3rd measurement**	13.21 ± 3.93 (12)^A^	13.84 ± 5.11 (12.9)^A^	13.08 ± 3.79 (12.6)^A^	.771
	**4th measurement**	13.28 ± 3.42 (13)^A^	14.10 ± 5.68 (12.5)^A^	13.23 ± 4.40 (13.6)^A^	.717
	**^2^ *p***	.914	.991	.849	
	**2nd–3rd**	**0**.**02 ± 5**.**92 (0**.**33)**	**‐0**.**11 ± 7**.**85 (−0**.**20)**	**0**.**03 ± 6**.**29 (−0**.**87)**	.996
	**3rd–4th**	**‐0**.**07 ± 5**.**24 (0**.**27)**	**‐0**.**26 ± 7**.**79 (−0**.**30)**	**−0**.**15 ± 6**.**59 (−2**.**08)**	.994

*Note*: ANOVA test, repeated measure ANOVA test. Different capital letters in the lines indicate the difference between groups.

Abbreviations: BS, bilateral stimulation; BSS, bilateral sham stimulation; SD, standard deviation, US, unilateral stimulation.

*p < .05.

Lactic acid values were compared between groups by taking the differences between the 2nd and 3rd measurements and the 3rd and 4th measurements and are shown in Table 3. The difference between the 2nd and 3rd measurements was seen the most in the BS group. When the difference in lactic acid values between the 3rd and 4th measurements is examined, the biggest difference belongs to the BSS group (6.21). The differences between the 2nd and 3rd measurements and the difference between the 3rd and 4th measurements show statistical significance according to the groups (*p* < .001) (Table 3).

The differences between the 2nd and 3rd measurements and the 3rd and 4th measurements of distance values show significant differences according to the groups (*p* < .001) (Table 3).

RMSSD and PNN50 values of the BS group increased significantly compared to the US and BSS groups in the evaluation made after the VSS application and at the 24th hour after the VSS application. In addition, after the VSS application, the RMSSD and PNN50 values of the US group increased significantly compared to the BSS group (Table 4).

**TABLE 4:** Comparison of intragroup, intergroup, and differences of parameters of the autonomic nervous system.

Parameters		Mean ± SD			^1^ *p*
		US	BS	BSS	
**RMSSD**	**1st measurement**	25.65 ± 9.51^A^	24.07 ± 9.75^A^	27.22 ± 8.5^A^	.425
	**2nd measurement**	7.25 ± 2.09^A^	7.31 ± 2.08^A^	7.45 ± 2.06^A^	.930
	**3rd measurement**	48.84 ± 6.95^A^	72.17 ± 15.08^B^	30.78 ± 5.48^C^	<.001
	**4th measurement (24th hour)**	23.56 ± 10.09^A^	30.11 ± 10.55^B^	20.56 ± 10.2^A^	.002
	**^2^ *p***	.001*	.001*	.001*	
	**2nd–3rd**	**−41**.**59 ± 7**.**54 (−41**.**76)**	**−64**.**86 ± 15**.**17 (−62**.**99)**	**−23**.**32 ± 5**.**47 (−24**.**32)**	**<**.**001**
	**3rd–4th**	**25**.**29 ± 10**.**24 (−25**.**34)**	**42**.**05 ± 17**.**72 (−35**.**85)**	**10**.**22 ± 12**.**77 (−10**.**56)**	**<**.**001**
**PNN50**	**1st measurement**	7.8 ± 8.86 (3.5)^A^	6.2 ± 7.03 (4)^A^	7.57 ± 7.03 (7)^A^	.686
	**2nd measurement**	0.7 ± 0.65 (1)^A^	0.8 ± 0.66 (1)^A^	0.7 ± 0.65 (1)^A^	.793
	**3rd measurement**	28.6 ± 9.28 (29.5)^A^	45.77 ± 14.34 (46.5)^B^	11.33 ± 5.33 (12)^C^	<.001
	**4th measurement (24th hour)**	5.15 ± 5.15 (4)^A^	11.6 ± 8.66 (10.5)^B^	3.93 ± 4.86 (2)^A^	<.001
	**^2^ *p***	.001*	.001*	.001*	
	**2nd–3rd**	**−27**.**90 ± 9**.**23 (−29)**	**−44**.**97 ± 14**.**46 (−46**.**5)**	**−10**.**63 ± 5**.**36 (−27**.**9)**	**<**.**001**
	**3rd–4th**	**23**.**45 ± 8**.**87 (−24**.**50)**	**34**.**17 ± 18**.**08 (−35)**	**7**.**40 ± 7**.**92 (−8)**	**<**.**001**
***LF power***	**1st measurement**	687.79 ± 490.99 (573.4)^A^	890.08 ± 931.73 (674)^AB^	1329.69 ± 1068.29 (830.7)^B^	.016
	**2nd measurement**	3106.02 ± 529.22 (3153.5)^A^	2831.11 ± 500.71 (2716.7)^A^	2997.71 ± 1106.84 (3131.3)^A^	.378
	**3rd measurement**	2102.65 ± 319.73 (2157.6)^A^	1246.37 ± 302.85 (1254.8)^B^	1726.53 ± 1046.27 (1440.7)^B^	<.001
	**4th measurement (24th hour)**	880.08 ± 965.93 (413.9)^A^	673.54 ± 500.49 (497)^A^	838.63 ± 708.12 (688.1)^A^	.531
	**^2^ *p***	.001*	.001*	.001*	
	**2nd–3rd**	**1003**.**37 ± 406**.**65 (1143**.**15)**	**1584**.**75 ± 349**.**63 (1557**.**88)**	**1271**.**18 ± 825**.**83 (1101**.**01)**	**<**.**001**
	**3rd–4th**	**1222**.**57 ± 978**.**41 (1406**.**20)**	**572**.**83 ± 539**.**48 (−720**.**54)**	**887**.**90 ± 1229**.**58 (−752**.**03)**	.**036**
**HF power**	**1st measurement**	312.91 ± 216.62 (260.3)^A^	329.45 ± 395.51 (235.8)^A^	407.87 ± 347.44 (263.3)^A^	.492
	**2nd measurement**	39.14 ± 22.83 (35.9)^A^	36.43 ± 18.22 (33.4)^A^	56.99 ± 27.89 (57.9)^B^	.002
	**3rd measurement**	296.95 ± 145.25 (286.4)^A^	532.66 ± 348.75 (460.1)^B^	68.61 ± 28.26 (67.7)^C^	<.001
	**4th measurement (24th hour)**	230.06 ± 183.42 (207.2)^AB^	384.18 ± 321.83 (327.8)^B^	159.85 ± 185.31 (97.9)^B^	.002
	**^2^ *p***	.001*	.001*	.001*	
	**2nd–3rd**	**−257**.**81 ± 146**.**78 (−249**.**93)**	**−496**.**23 ± 348**.**23 (−416**.**73)**	**−11**.**62 ± 4**.**19 (−10**.**43)**	**<**.**001**
	**3rd–4th**	**66**.**88 ± 229**.**79 (−102**.**72)**	**148**.**48 ± 384**.**47 (−139**.**77)**	**−91**.**25 ± 196**.**65 (21**.**10)**	.**005**
***Lf/Hf ratio***	**1st measurement**	3.5 ± 4.21 (1.7)^A^	4.17 ± 3.55 (3.4)^A^	4.25 ± 3.52 (3.3)^A^	.704
	**2nd measurement**	113.27 ± 76.47 (87.2)^A^	95.58 ± 53.21 (76.2)^A^	68.58 ± 49.48 (53.3)^B^	.020
	**3rd measurement**	9.8 ± 9.93 (6.8)^A^	2.84 ± 1.15 (2.7)^B^	31.75 ± 27.67 (26.3)^C^	<.001
	**4th measurement (24th hour)**	3.84 ± 2.62 (3.4)^A^	2.08 ± 1.24 (1.8)^B^	8.92 ± 6.34 (6.4)^C^	<.001
	**^2^ *p***	.001*	.001*	.001*	
	**2nd–3rd**	**103**.**47 ± 76**.**64 (73**.**57)**	**92**.**74 ± 53**.**33 (74**.**05)**	**36**.**83 ± 41**.**61 (24**.**07)**	**<**.**001**
	**3rd–4th**	**−5**.**97 ± 9**.**86 (−3**.**76)**	**−0**.**75 ± 1**.**87 (−0**.**86)**	**−22**.**84 ± 28**.**89 (−17**.**17)**	**<**.**001**

*Note*: ANOVA test, repeated measure ANOVA test. Different capital letters in the lines indicate the difference between groups.

Abbreviations: BS, bilateral stimulation; BSS, bilateral sham stimulation; SD, standard deviation, US, unilateral stimulation.

*p < .05.

After the VSS application, the high frequency (HF) values of the BS and US groups increased significantly compared to the BSS group. In addition, the HF values of the BS group increased significantly compared to the US group. In the evaluation made at the 24th hour after the VSS application, the HF values of the BS group increased significantly compared to the BSS group. However, no significant difference was found between the other groups (Table 4).

Considering the intragroup changes in the evaluation made after the VSS application and at the 24th hour after the VSS application, RMSSD, PNN50, and HF values increased significantly in all groups. After the VSS application, the LF values of the BS and BSS groups decreased significantly compared to the US group. However, there was no significant difference between the other groups and in the evaluation made at the 24th hour after the VSS application (Table 4).

In the evaluation made after the VSS application and at the 24th hour after the VSS application, the LF/HF ratio values of the BS and US groups decreased significantly compared to the BSS group. In addition, the LF/HF ratio values of the BS group were significantly lower than the US group. Considering the intragroup changes in the evaluation made after the VSS application and at the 24th hour after the application, LF and LF/HF ratio values decreased significantly in all groups (Table 4).

The differences in RMSSD values between the 2nd and 3rd measurements and the 3rd and 4th measurements show significant differences according to the groups (*p* < .001). The differences in PNN50 values between the 2nd and 3rd measurements and the 3rd and 4th measurements show significant differences according to the groups (*p* < .001). The differences between the 2nd and 3rd measurements of LF and HF powers values show significant differences according to the groups (*p* < .001). The differences between the 2nd and 3rd measurements of LF/HF Ratio values show significant differences according to the groups (*p* < .001) (Table 4).

After VSS application on all days, the pulse and systolic pressure values of the US and BS groups decreased significantly compared to the BSS group. On all days, the heart rate and systolic pressure values of the BS group (except the 1st day) were significantly lower than the US group. In addition, diastolic pressure values of the BS group decreased significantly compared to the BSS group after VSS application on all days (Table 5).

**TABLE 5:** Comparison of intragroup, intergroup, and differences in blood pressure and pulse parameters.

			Mean ± SD			^1^ *p*
			US	BS	BSS	
**Pulse**	**1st day**	**1st measurement**	88.73 ± 12.84^A^	88.3 ± 12.38^A^	94.2 ± 14.78^A^	.169
		**2nd measurement**	131.03 ± 10.02^A^	132.0 ± 8.18^AB^	137.20 ± 8.42^B^	.019
		**3rd measurement**	109.30 ± 10.28^A^	109.93 ± 6.13^A^	120.40 ± 10.24^B^	<.001
		**^2^ *p***	.001*	.001*	.001*	
		**2nd–3rd**	**21**.**73 ± 4**.**22 (23)**	**22**.**07 ± 2**.**83 (23)**	**16**.**80 ± 8**.**91 (18**.**5)**	**<**.**001**
	**2nd day**	**1st measurement**	95.23 ± 9.96^AB^	89.23 ± 12.69^A^	96.87 ± 11.42^B^	.028
		**2nd measurement**	132.83 ± 8.24^A^	131.10 ± 6.38^A^	135.4 ± 9.70^A^	.131
		**3rd measurement**	110.03 ± 9.85^A^	104.17 ± 5.58^B^	119.87 ± 8.93^C^	<.001
		**^2^ *p***	.001*	.001*	.001*	
		**2nd–3rd**	**22**.**80 ± 5**.**05 (24**.**5)**	**26**.**93 ± 2**.**41 (28)**	**15**.**53 ± 8**.**67 (19**.**5)**	**<**.**001**
	**3rd day**	**1st measurement**	94.63 ± 9.94^A^	89.13 ± 13.64^A^	92.10 ± 13.29^A^	.234
		**2nd measurement**	134.33 ± 8.88^A^	130.97 ± 9.32^A^	135.97 ± 9.55^A^	.109
		**3rd measurement**	109.67 ± 8.41^A^	104.03 ± 7.58^B^	117.90 ± 8.26^C^	<.001
		**^2^ *p***	.001*	.001*	.001*	
		**2nd–3rd**	**24**.**67 ± 2**.**81 (25)**	**26**.**93 ± 2**.**96 (28)**	**18**.**07 ± 5**.**13 (19**.**5)**	**<**.**001**
	**4th day**	**1st measurement**	91.83 ± 12.01^A^	88.63 ± 11.27^A^	93.80 ± 9.50^A^	.190
		**2nd measurement**	133.37 ± 8.23^A^	129.27 ± 7.83^A^	134.07 ± 10.63^A^	.088
		**3rd measurement**	109.17 ± 8.97^A^	101.63 ± 6.05^B^	115.83 ± 10.54^C^	<.001
		**^2^ *p***	.001*	.001*	.001*	
		**2nd–3rd**	**24**.**20 ± 1**.**85 (24)**	**27**.**63 ± 2**.**71 (28)**	**18**.**23 ± 6**.**89 (18)**	**<**.**001**
**Systolic blood pressure**	**1st day**	**1st measurement**	125.4 ± 14.43^A^	126.37 ± 7.95^A^	126.17 ± 11.97^A^	.945
		**2nd measurement**	163.3 ± 8.33^A^	164.33 ± 5.56^A^	164.67 ± 9.49^A^	.787
		**3rd measurement**	142.17 ± 7.97^A^	133.87 ± 5.37^B^	153.57 ± 9.27^C^	<.001
		**^2^ *p***	.001*	.001*	.001*	
		**2nd–3rd**	**21**.**13 ± 0**.**94 (21)**	**30**.**47 ± 1**.**20 (30**.**5)**	**11**.**10 ± 1**.**12 (11)**	**<**.**001**
	**2nd day**	**1st measurement**	124.07 ± 6.2^A^	122.57 ± 8.97^A^	126.67 ± 8.63^A^	.141
		**2nd measurement**	162.6 ± 5.17^A^	162.0 ± 6.55^A^	165.27 ± 5.08^A^	.063
		**3rd measurement**	141.67 ± 5.66^A^	131.9 ± 6.45^B^	153.97 ± 4.92^C^	<.001
		**^2^ *p***	.001*	.001*	.001*	
		**2nd–3rd**	**20**.**93 ± 1**.**08 (21)**	**30**.**10 ± 1**.**63 (31)**	**11**.**30 ± 0**.**84 (12)**	**<**.**001**
	**3rd day**	**1st measurement**	124.13 ± 7.32^A^	122.4 ± 8.25^A^	126.33 ± 8.25^A^	.164
		**2nd measurement**	163.47 ± 5.49^A^	161.6 ± 6.4^A^	163.83 ± 4.99^A^	.266
		**3rd measurement**	142.6 ± 5.52^A^	131.23 ± 6.17^B^	152.7 ± 4.89^C^	<.001
		**^2^ *p***	.001*	.001*	.001*	
		**2nd–3rd**	**20**.**87 ± 1**.**11 (21)**	**30**.**37 ± 1**.**45 (31)**	**11**.**13 ± 0**.**82 (11)**	**<**.**001**
	**4th day**	**1st measurement**	124.4 ± 8.24^A^	121.5 ± 8.53^A^	126.27 ± 8.5^A^	.093
		**2nd measurement**	163.23 ± 6.07^AB^	160.37 ± 6.35^A^	164.1 ± 5.51^B^	.046
		**3rd measurement**	142.3 ± 6.24^A^	129.8 ± 6.14^B^	152.6 ± 5.77^C^	<.001
		**^2^ *p***	.001*	.001*	.001*	
		**2nd–3rd**	**20**.**93 ± 1**.**11 (21)**	**30**.**57 ± 1**.**74 (31)**	**11**.**50 ± 1**.**01 (12)**	**<**.**001**
**Diastolic blood pressure**	**1st day**	**1st measurement**	84.3 ± 13.87^A^	83.1 ± 11.11^A^	84.03 ± 10.52^A^	.920
		**2nd measurement**	94.87 ± 11.85^A^	93.8 ± 9.92^A^	96.5 ± 10.37^A^	.620
		**3rd measurement**	88.67 ± 11.84^AB^	85.53 ± 10.05^A^	92.77 ± 10.48^B^	.039
		**^2^ *p***	.001*	.001*	.001*	
		**2nd–3rd**	**6**.**20 ± 0**.**55 (6)**	**8**.**27 ± 0**.**45 (8)**	**3**.**73 ± 0**.**87 (3)**	**<**.**001**
	**2nd day**	**1st measurement**	84.1 ± 10.06^A^	80.33 ± 10.08^A^	82.33 ± 12.49^A^	.414
		**2nd measurement**	94.87 ± 8.74^A^	91.27 ± 9.49^A^	94.67 ± 12.27^A^	.318
		**3rd measurement**	88.37 ± 8.5^AB^	82.67 ± 9.56^A^	90.9 ± 11.93^B^	.007
		**^2^** *p*	0.001*	0.001*	0.001*	
		**2nd–3rd**	**6**.**50 ± 0**.**63 (6)**	**8**.**60 ± 0**.**81 (8)**	**3**.**77 ± 0**.**82 (4)**	**<**.**001**
	**3rd day**	**1st measurement**	81.77 ± 11.3^A^	78.57 ± 8.34^A^	83.87 ± 9.52^A^	.114
		**2nd measurement**	94.07 ± 9.37^A^	91.37 ± 8.02^A^	94.63 ± 9.12^A^	.316
		**3rd measurement**	86.43 ± 9.28^AB^	81.5 ± 7.63^A^	90.97 ± 9^B^	<.001
		**^2^ *p***	.001*	.001*	.001*	
		**2nd–3rd**	**7**.**63 ± 2**.**11 (7**.**5)**	**9**.**87 ± 0**.**78 (10)**	**3**.**67 ± 0**.**96 (3)**	**<**.**001**
	**4th day**	**1st measurement**	80.93 ± 8.86^A^	78.03 ± 9.56^A^	83.47 ± 9.22^A^	.079
		**2nd measurement**	92.4 ± 7.18^A^	91.3 ± 8.87^A^	93.67 ± 9.15^A^	.556
		**3rd measurement**	85.63 ± 6.68^AB^	81.47 ± 8.58^A^	90.3 ± 9.16^B^	<.001
		**^2^ *p***	.001*	.001*	.001*	
		**2nd–3rd**	**6**.**77 ± 2**.**03 (7)**	**9**.**83 ± 1**.**02 (10)**	**3**.**37 ± 0**.**67 (3)**	**<**.**001**

*Note*: ANOVA test, repeated measure ANOVA test. Different capital letters in the lines indicate the difference between groups.

Abbreviations: BS, bilateral stimulation; BSS, bilateral sham stimulation; SD, standard deviation, US, unilateral stimulation.

*p < .05.

In the evaluation made at the 24th hour after the 1st day VSS application, the pulse values of the BS group were significantly lower than the BSS group. However, there was no significant difference between the other groups and in the 2nd day/3rd/4th day evaluation (Table 5).

Considering the changes within the group after the VSS application, pulse and systolic/diastolic pressure values decreased significantly in all groups on all days (Table 5).

The difference between the pulse value on the 1st day, the 2nd measurement, and the 3rd measurement shows a statistically significant difference between the groups. The largest decrease among the 1st day, 2nd, and 3rd measurement was seen in the BS group (*p* < .001). The difference in pulse value between the 2nd and the 3rd measurements on the 2nd day shows a statistically significant difference between the groups. The largest decrease among the 2nd day, 2nd, and 3rd measurement was seen in the BS group (*p* < .001). When the pulse values were examined, the greatest decrease between the 2nd and 3rd measurements was seen in the BS group in the measurements made in all 4 days. Similarly, when the pulse values were examined, the highest decrease between the 2nd and 3rd measurements in the measurements made in 4 days was seen in the BS group. The decrease between the 2nd and 3rd measurement shows a statistically significant difference between the groups (*p* < .001) (Table 5).

The difference in systolic blood pressure value between the 2nd and the 3rd measurements on the 1st day shows a statistically significant difference between the groups. The largest decrease among the 1st day, 2nd, and 3rd measurement was seen in the BS group (*p* < .001). The difference in systolic blood pressure value between the 2nd and the 3rd measurements on the 2nd day shows a statistically significant difference between the groups. The largest decrease among the 2nd day, 2nd, and 3rd measurement was seen in the BS group (*p* < .001). When the systolic blood pressure values were examined, the highest decrease between the 2nd and 3rd measurements was seen in the BS group in the measurements made on all 4 days. Similarly, when systolic blood pressure values were examined, the highest decrease between the 2nd and 3rd measurements in the measurements made in 4 days was seen in the BS group. The decrease between the 2nd and 3rd measurements shows a statistically significant difference between the groups (*p* < .001) (Table 5).

The difference in diastolic blood pressure value between the 2nd and 3rd measurements on the 1st day shows a statistically significant difference between the groups. The largest decrease among the 1st day, 2nd, and 3rd measurement was seen in the BS group (*p* < .001). The difference in diastolic blood pressure value between the 2nd and the 3rd measurements on the 2nd day shows a statistically significant difference between the groups. The largest decrease among the 2nd day, 2nd, and 3rd measurement was seen in the BS group (*p* < .001). When diastolic blood pressure values were examined, the highest decrease between the 2nd and 3rd measurements was seen in the BS group in the measurements made on all 4 days. Similarly, when diastolic blood pressure values were examined, the highest decrease between the 2nd and 3rd measurements in the measurements made in 4 days was seen in the BS group. The decrease between the 2nd and 3rd measurement shows a statistically significant difference between the groups (*p* < .001) (Table 5).

## 4 DISCUSSION

In our study, the effect of transcutaneous auricular VNS applied for 4 days on healthy young individuals on pain, fatigue, blood pressure, heart rate, distance covered, lactic acid level, and autonomic nervous system activity was investigated. Volunteers participating in the study were randomly divided into three groups as unilateral left stimulation, BS, and bilateral sham stimulation with equal numbers of males and females. Overall, we can argue that auricular VNS can reduce pain and fatigue and accelerate recovery by reducing exercise‐induced sympathetic hyperactivity. We can also think that BS is superior to unilateral left stimulation, as seen in the decrease in lactic acid levels. We found that the changes in blood pressure and pulse with auricular VNS paralleled the changes in autonomic activity, and that VNS (left or bilateral) did not cause bradycardia or hypotension. With all these, we saw that VNS did not increase cycling distance.

Today, sport is one of the most important social activities that is followed with great interest by a wide audience and performed by most of us personally. Even in today's world countries, sports have become the way for countries to show strength to each other. As such, it is not just an athlete who is involved in physical activity. In recent years, professional athletes in all branches of sports have a very intense training and match schedule. For example, in the US National Basketball League, it is seen that athletes play every 2 days in some periods. When football clubs are considered, when the league, European, and cup matches come to the same period, the athletes play in periods of 2–3 days. As a result of this intense calendar, the recovery of the athletes cannot reach the desired level. Inadequate recovery, on the other hand, reduces performance in the long run and can lead to injuries in addition to this. Parasympathetic capacity is the determinant of restructuring and recovery (restoration) after exercise (Chen et al., 2011). During the recovery period of the person, the sympathetic system activity is suppressed, and the parasympathetic system activity becomes prominent. Autonomic nervous system regulation after exercise, by accelerating the parasympathetic activity return, can increase recovery in athletes and can be practically done with noninvasive VNS. There is a very limited evidence in the literature examining the effect of VNS on recovery and performance in sports (Coote, 2010; Bucheit et al., 2007; Pugh & Pugh, 2021). In this study, we aimed to investigate the potential benefits and effects of auricular VNS on sportive activity, in healthy young people through cycling exercise.

Cook et al. (2004) used a numerical pain scale to evaluate pain after cycling exercise in 16 African‐American women with a history of parental hypertension. In the study of Jameson and Ring in which they compared the effect of different wattage and cadence of cycling exercise, they evaluated pain with a numerical pain scale (Jameson & Ring, 2000). In the study evaluating the pain caused by the maximum and sub‐maximum cycling exercise in the leg muscle, it was compared with the numerical pain scale (Motl et al., 2007). In our study, numerical pain scale was used too as the evaluation method and BS was superior to US, which in turn was superior to BSS, in reducing pain after exercise.

Studies in the literature evaluate pain perception together with heart rate, systolic, and diastolic blood pressure (Koltyn & Arbogast, 1998). Vascular function and blood analysis are the other methods for exercise research studies (Alkatan et al., 2016). We analyzed participants’ pulse, systolic, and diastolic blood pressure to monitor as side effects and did not encounter bradycardia and hypotension in any group. At the same time, BS was superior to US, which in turn was superior to BSS to normalize pulse and blood pressure after exercise.

In a study consisting of 18 healthy adult male geneticists, the level of fatigue was regularly questioned during cycling exercise and rest, and a numerical fatigue scale was used for this (Micklewright et al., 2017). Another study found that high‐intensity cardiorespiratory and strength exercises reduced fatigue and sensory distress in patients with axial spondyloarthritis. Numerical fatigue scale was used to evaluate fatigue in the study (Sveaas et al., 2018). In our study, the fatigue level of the participants was evaluated with a numerical fatigue scale. We found similar results for fatigue perception as for pain, with the strongest effect in the BS group.

A total of 14 healthy individuals participated in a study investigating the effect of HRV on biofeedback, autonomic function, and functional connectivity of the prefrontal cortex. Autonomic nervous system was evaluated with the Polar H10 device and the Elite HRV program (Schumann et al., 2020). The effect of training load applied at different levels on HRV and running performance was investigated in the study, which included seven athletes in the Olympic level rugby team. Polar H7 device and Elite HRV program were used to evaluate HRV and autonomic nervous system (Flatt & Howells, 2019). Similar to the examples in the literature, Polar H10 device and Elite HRV program were used in our study. Different parameters depending on the analysis of the variability between heartbeat times can be used in the evaluation of autonomic nervous system activity. One of these, HF power, which indicates parasympathetic system activity, may reflect insufficient recovery from previous training, and this may indicate unfavorable conditions for performance improvements (Chalencon et al., 2012). In the recovery period after exercise, parasympathetic nervous system activity occupies a very important place to reestablish homeostasis (Javorka et al., 2002). We measured HRV to evaluate the status of the autonomic nervous system branches and saw that BS was superior to US, which in turn was superior to BSS in increasing parasympathetic nervous system activity and decreasing sympathetic nervous system activity. Although studies comparing bilateral and US are rare, bilateral is more likely to be effective (Özden, 2023).

The lactate scout device was used in the study evaluating the lactate concentration in the blood of 17 Polish sprinters competing in the elite 100 m category (Kawczyński et al., 2015). A total of 14 people participated in the study investigating the acute effect of peristaltic pneumatic compression. In the study, the repeated anaerobic exercise performance of the participants and the level of lactate in the blood were investigated. The lactate level in the blood was evaluated with the Lactate scout device (Martin et al., 2015). In our study, this device was used to examine the lactate level. BS decreased lactic acid level and increased parasympathetic activity, and these results seem consistent with the study of Conrado de Freitas et al. They stated that increased blood lactate concentrations and decreased recovery of parasympathetic modulation are interrelated (Freitas et al., 2021).

Uthman et al. published a 12‐year observation in their study investigating the effectiveness of VNS in patients with epilepsy. A total of 28 people with persistent epilepsy participated in the study. The VNS was performed using a device implanted in the neck. Seizure numbers were noted over the 12‐year observation period from the initial moment. The side effects of the application were found to be mild to moderate. The mean seizure frequency was found to decrease by 26% after 1 year, 30% after 5 years, and 52% after 12 years with VNS (Uthman et al., 2004). Rush et al. investigated the effect of VNS on treatment‐resistant depression. In this multicenter study, 30 patients who did not benefit from at least two drug trials were included. The application was made with a device implanted in the neck while using the drug. The study, in which the control group was included, lasted 10 weeks. In the study, it was concluded that VNS has as much effects as antidepressants in treatment‐resistant depression (Rush et al., 2000). VNS can also be used for other diseases like treatment‐resistant anxiety. A 50% or greater improvement in the Hamilton anxiety scale for all patients and 25% or greater improvement in the Yale‐Brown obsessive‐compulsive scale for patients with obsessive‐compulsive disorder were seen in the literature (George et al., 2008). VNS has been used in many diseases, and its use tends to expand as its success increases. Transcutaneous auricular VNS is a safe and feasible method (Kim et al., 2022). Therefore, we chose this application in our study and did not see any side effects in the participants.

A total of 16 people participated in Koltyn et al.’s study evaluating pain perception following aerobic exercise. As an exercise, a bicycle ergometer was used for 30 min. A 30‐min rest period was applied after the exercise. In the study, pain was evaluated with a numerical pain scale and an algometer. There was an increase in pain perception after an acute exercise. However, there was a decrease in pain perception during exercise compared to the rest period (Koltyn & Arbogast, 1998). In our study, the second measurement value was higher than the first measurement value every day in all groups. This is due to the increased perception of pain after exercise, and our result is similar to the literature.

There are research studies about VNS effect on pain. Bush et al. conducted an experimental study investigating the effect of transcutaneous VNS on pain perception. It was evaluated with a quantitative sensory test including the tonic heat pain paradigm in 48 healthy volunteers. Each subject attended two experimental sessions in a random order with stimulation or sham stimulation on different days. The stimulated group significantly reduced the level of pain during continuous application of painful heat for 5 min compared to the sham group. No related changes in cardiac or respiratory activity or clinically relevant adverse effects were observed during administration (Busch et al., 2013). In another study, 10 healthy volunteers participated for the effect of VNS applied from the left cervical region on pain. Pain is created by heat, pressure, and short‐term pulsing. Pain was evaluated with a visual analog scale. As a result of the study, it was found that VNS was effective in reducing pain in humans (Kirchner et al., 2000). In our study, in all groups, the 3rd measurement value is lower than the 2nd measurement value. This shows that pain decreased in all groups. However, the values in the US and BSS groups are higher than those in the BS group. In other words, VSS is effective in relieving pain. However, when we compare the BSS and US groups, there is no difference in the effect on pain.

De Souza et al. investigated the acute effects of two types of aerobic exercise on maximum strength and endurance. Eight men with a high level of physical activity participated. It has a randomized design in which all subjects complete both control and experimental conditions. Each session was held 1 week apart. The study revealed that both aerobic and strength exercises produced significant peripheral fatigue in the same muscle group (De Souza et al., 2007). In a study investigating the acute effect of aerobic exercise on mood, 32 female students aged 18–23 participated in a single‐session experiment in which they performed two 8‐min high‐intensity exercise trials and two 8‐min low‐intensity exercise trials. Fatigue level was evaluated with Borg fatigue scale. In general, they found that high‐intensity exercise leads to an increase in tension/anxiety and fatigue (Steptoe & Cox, 1988). In our study, the second measurement values of the participants in all groups were higher than the first measurement values. As in the literature, we have observed that exercise acutely increases fatigue.

Another study was conducted investigating the effect of noninvasive VNS on fatigue and immune responses in patients with primary Sjögren's syndrome. The VNS was applied from the cervical region in a noninvasive manner, twice daily over a 26‐day period. They found that the vagus nerve may play a role in the regulation of fatigue and immune responses in patients with primary Sjögren's syndrome and may reduce the clinical symptoms of VNS related to fatigue and sleep problems (Tarnet al., 2019). VNS can also be effective for insomnia. Jiao et al. mentioned that VNS relieves fatigue and improves participants’ quality of life and other accompanying symptoms such as depression and anxiety (Jiao et al., 2020). Similar to the literature, as in our study, fatigue scores obtained from the BSS group were higher than the US and BS groups. This shows that VNS is effective in reducing fatigue. However, the superiority of the single and double ear application over each other was not observed.

## 5 CONCLUSION

There is limited evidence in the literature about VNS effect on exercise recovery and performance. After exercise, auricular VNS can decrease fatigue, pain, and lactic acid levels, increase parasympathetic activity without corruptive effects on pulse and blood pressure. Cycling distance did not change in our study, and this is probably because performance is related to motivation and ambition mostly. Moreover, BS seems more potent to unilateral and sham stimulation. We think that noninvasive VNS can be used in recovery practice in sports in the future. More studies are needed which should include intra‐subject designs, investigate physiological mechanisms with their complexity.

## 6 LIMITATIONS

The short follow‐up period and the lack of long‐term application of VNS can be noted as limitations. Additionally, a real sham group may not have been fully formed. Our study was a between‐group design and not a within‐group design.

## CONFLICT OF INTEREST STATEMENT

The authors declare that they have no known conflicts of interest or personal relationships that could have appeared to influence the work reported in this paper.The authors contributed equally to the article. Final draft by all authors.

## FUNDING INFORMATION

Not applicable.

### PEER REVIEW

The peer review history for this article is available at https://publons.com/publon/10.1002/brb3.3332.

## Data Availability

The authors do not have permission to share data.
